# Cholesterol depletion does not alter the capacitance or Ca handling of the surface or t‐tubule membranes in mouse ventricular myocytes

**DOI:** 10.14814/phy2.13500

**Published:** 2017-11-17

**Authors:** Hanne C. Gadeberg, Cherrie H. T. Kong, Simon M. Bryant, Andrew F. James, Clive H. Orchard

**Affiliations:** ^1^ School of Physiology, Pharmacology & Neuroscience Biomedical Sciences Building University of Bristol Bristol United Kingdom

**Keywords:** Cholesterol, methyl‐*β*‐cyclodextrin, t‐tubules

## Abstract

Cholesterol is a key component of the cell plasma membrane. It has been suggested that the t‐tubule membrane of cardiac ventricular myocytes is enriched in cholesterol and that this plays a role in determining t‐tubule structure and function. We have used methyl‐*β*‐cyclodextrin (M*β* CD) to deplete cholesterol in intact and detubulated mouse ventricular myocytes to investigate the contribution of cholesterol to t‐tubule structure, membrane capacitance, and the distribution of Ca flux pathways. Depletion of membrane cholesterol was confirmed using filipin; however, di‐8‐ANEPPS staining showed no differences in t‐tubule structure following M*β* CD treatment. M*β* CD treatment had no significant effect on the capacitance:volume relationship of intact myocytes or on the decrease in capacitance:volume caused by detubulation. Similarly, Ca influx and efflux were not altered by M*β* CD treatment and were reduced by a similar amount following detubulation in untreated and M*β* CD‐treated cells. These data show that cholesterol depletion has similar effects on the surface and t‐tubule membranes and suggest that cholesterol plays no acute role in determining t‐tubule structure and function.

## Introduction

Cholesterol is a key component of the cell plasma membrane. It has been suggested that the t‐tubules of cardiac ventricular myocytes – invaginations of the surface membrane that play a central role in excitation‐contraction coupling – are enriched in cholesterol compared with the surface membrane (Pásek et al. 2008), and that cholesterol is involved in t‐tubule formation and maintenance (Carozzi et al. 2000); it has recently been reported that depleting membrane cholesterol disrupts the t‐tubule network of cardiac myocytes (Zhu et al. 2016), although previous work has shown that cholesterol depletion has no effect on t‐tubule morphology (Calaghan and White 2006).

Membrane cholesterol is concentrated in lipid rafts and caveolae, specialized areas of the cell membrane that play an important role in the localization of individual proteins and macromolecular assemblies of signaling complexes (reviewed in Balijepalli and Kamp 2008) many of which, such as Ca_v_1.2 (which carries the L‐type Ca current, I_Ca_) and the *β*_2_ adrenergic pathway, are located predominantly in the t‐tubules (Bryant et al. 2014).

Inhomogeneous distribution of cholesterol between the surface and t‐tubule membranes may also have important implications for the study of t‐tubules because cholesterol content alters membrane capacitance (Pásek et al. 2008). Many studies investigating t‐tubule function have used detubulation (physical and functional uncoupling of t‐tubules from the surface membrane; Kawai et al. 1999; Brette et al. 2002). If the cholesterol content, and thus capacitance, of the t‐tubule membrane is different from that of the surface membrane, the loss of membrane capacitance following detubulation may not accurately reflect the loss of membrane area. This would alter the calculated t‐tubule membrane fraction and thus t‐tubular density of membrane currents, and this could account for the differences in t‐tubule membrane fraction obtained using imaging and electrophysiological techniques.

We have, therefore, investigated the effect of cholesterol depletion, using methyl‐*β*‐cyclodextrin (M*β*CD), on the decrease in membrane capacitance, and on the distribution of Ca flux via I_Ca_ and Na/Ca exchange current (I_NCX_), obtained using detubulation. This enabled us to assess directly whether cholesterol depletion alters t‐tubular membrane capacitance or Ca flux, in contrast to previous measurements in intact cells, in which t‐tubular changes may be masked by reciprocal changes at the cell surface, as reported recently in heart failure (Bryant et al. 2015); this may be important if cholesterol is involved in localization of proteins, and thus Ca flux, at the t‐tubules, in which case cholesterol depletion might lead to protein redistribution.

## Materials and Methods

### Myocyte isolation, detubulation, and methyl‐β‐cyclodextrin treatment

Mouse ventricular myocytes were isolated from the hearts of C57BL/6 mice aged between 11 and 13 weeks as described previously (Gadeberg et al. 2017). All procedures were performed in accordance with UK legislation, approved by the University of Bristol Ethics Committee, and reported in accordance with the ARRIVE guidelines. Following isolation, cells were kept in storage solution containing (in mmol/L): 130 NaCl, 5.4 KCl, 0.4 NaH_2_PO_4_, 4.2 HEPES, 10 Glucose, 1.4 MgCl_2_, 20 taurine, 10 creatine, 0.1 CaCl_2_, pH 7.4 (with NaOH).

Cells were detubulated using formamide‐induced osmotic shock by incubating the cells with 1.5 mol/L formamide for 2 min before centrifugation and resuspending the cells in storage solution containing 1 mmol/L CaCl_2_.

Membrane cholesterol was depleted by incubating cells with 1 mmol/L methyl‐*β*‐cyclodextrin (M*β*CD; Sigma‐Aldrich, Poole, UK) in storage solution for 1 h at 37°C. For detubulated M*β*CD‐treated cells, cells were first detubulated before depleting them of cholesterol.

### Membrane staining

Membrane cholesterol was stained using filipin III (Sigma‐Aldrich, Poole, UK). Cells were fixed using 4% paraformaldehyde for 10 min before incubating in 0.05 mg/mL filipin in 10% fetal bovine serum for 2 h. Cells were mounted with Prolong Gold before imaging on a Leica SP8 tandem scanning system with a 1.1 numerical aperture 40 ×  water immersion objective. Filipin was excited with a Spectra Physics DeepSee tuneable multiphoton laser set to 760 nm and light collected between 451 and 515 nm.

Cell membrane staining was performed by incubating cells in 5 *μ*mol/L di‐8‐ANEPPS at room temperature. Cells were imaged on an LSM 880 confocal microscope (Zeiss, Germany) in Airyscan “super‐resolution” mode, with a 1.3 numerical aperture 63 ×  oil immersion objective, with voxel size set to 40 nm in‐plane and 180 nm along the optical axis.

Image analysis was performed using MATLAB R2015a (Mathworks, Inc.) and ImageJ (v1.50, NIH). The regularity of t‐tubule staining was quantified by applying a two‐dimensional (2D) Fast Fourier Transform (FFT) to a square region of the interior of the cell, and the power of the first harmonic normalized to that of the zeroth harmonic (P_1_/P_0_). For M*β*CD‐treated cells stained with filipin where no first harmonic was observed, the power was taken at the mean frequency of the first harmonic in untreated cells. T‐tubule density was calculated from the 3D skeleton of di‐8‐ANEPPS stained cells and normalized to volume.

### Electrophysiological recordings

Myocytes were placed in a chamber mounted on a Diaphot inverted microscope (Nikon UK Ltd, Kingston‐upon‐Thames, UK). Membrane currents and cell capacitance were recorded using the whole‐cell patch‐clamp technique, as described previously (Gadeberg et al. 2017).

To monitor Ca influx and efflux, the holding potential was set to −80 mV; a 500 msec ramp to ‐40 mV was used to inactivate I_Na_, followed by step depolarization to 0 mV for 100 msec to activate I_Ca_, at a frequency of 1 Hz. I_Ca_ was measured as the difference between peak inward current and current at the end of the pulse to 0 mV and the integral taken as a measure of Ca influx. I_NCX,tail_, the current representing Ca removed by NCX following the step depolarization, was measured by fitting a single exponential function to 350 msec of the current trace starting 20 msec after repolarization from 0 to −80 mV, and extrapolating back to when the membrane was repolarized. The integral of the exponential was taken as a measure of Ca efflux (Fedida et al. 1987; Giles and Shimoni 1989). This analysis was performed using Matlab R2015a. Both influx and efflux were normalized to cell volume, which was calculated from measurements of cell length and width as the volume of an elliptical cylinder, assuming a depth of 0.66 cell width (based on di‐8‐ANEPPS‐stained images).

### Statistics

Data are expressed as mean ± SEM; n is given as c/h, where c is the number of cells used from h hearts. Student's *t*‐test and 2‐way ANOVA with Bonferroni post hoc test were performed as appropriate using GraphPad Prism 7 (GraphPad Software Inc.) based on the number of cells. The limit of statistical confidence was *P* < 0.05.

## Results

### Cholesterol depletion and t‐tubule structure

**Figure 1:**
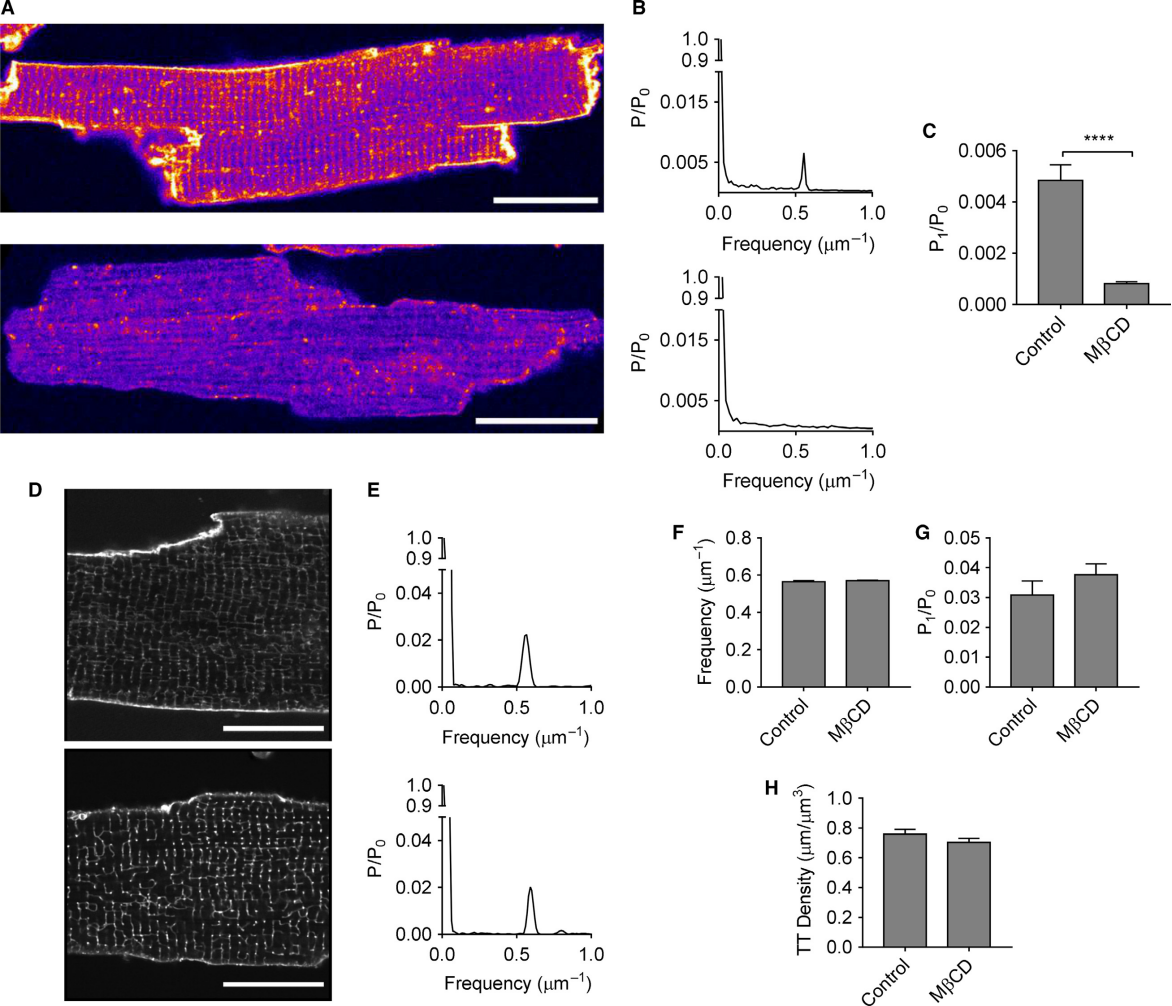
(A) Representative images of control (top) and M*β* CD‐treated (bottom) cells stained with filipin. Scalebars represent 25 *μ*m; (B) Corresponding power spectra. (C) Amplitude of the first harmonic of the filipin power spectra in control and M*β* CD‐treated cells (*n* = 16/3 and *n* = 14/3, respectively). (D) Representative confocal images of control (top) and M*β* CD‐treated (bottom) myocytes stained with di‐8‐ANEPPS. Scalebars represent 20 *μ*m. (E) Corresponding power spectra. (F) Mean frequency and (G) amplitude of the first harmonic of the di‐8‐ANEPPS power spectra. (H) Mean t‐tubule density. Control *n* = 14/3, M*β* CD *n* = 27/5, **** *P* < 0.0001 with Student's *t*‐test.

The effectiveness of M*β*CD treatment in removing membrane cholesterol was assessed by staining cells with filipin, a fluorescent probe which binds cholesterol. Figure 1A shows representative images of control (top) and M*β*CD‐treated (bottom) ventricular myocytes, showing cholesterol in the surface sarcolemma and t‐tubules of control myocytes, which was absent after treatment with M*β*CD. This was quantified using FFT analysis (Fig. 1B), which showed a first harmonic in untreated cells at a mean frequency of 0.57 ± 0.01 *μ*m^−1^ (*n* = 16/3), consistent with t‐tubule staining, but no peak in M*β*CD‐treated cells (*n* = 14/3; Fig. 1C). Thus, treatment with M*β*CD appears to be effective at depleting membrane, including t‐tubular, cholesterol, consistent with previous work (Carozzi et al. 2000; Agarwal et al. 2011; Odnoshivkina et al. 2017).

It has been suggested that cholesterol is required for t‐tubule integrity in mouse cells (Zhu et al. 2016). Myocytes were, therefore, stained with di‐8‐ANEPPS to determine the effect of treatment with M*β*CD on t‐tubule structure. Example images and power spectra are shown in Figure 1D and E, respectively. M*β*CD did not alter the regularity of the t‐tubule network, assessed using the frequency (Fig. 1F) and amplitude (Fig. 1G) of the first harmonic of the power spectrum, nor did it alter t‐tubule density (Fig. 1H).

**Figure 2:**
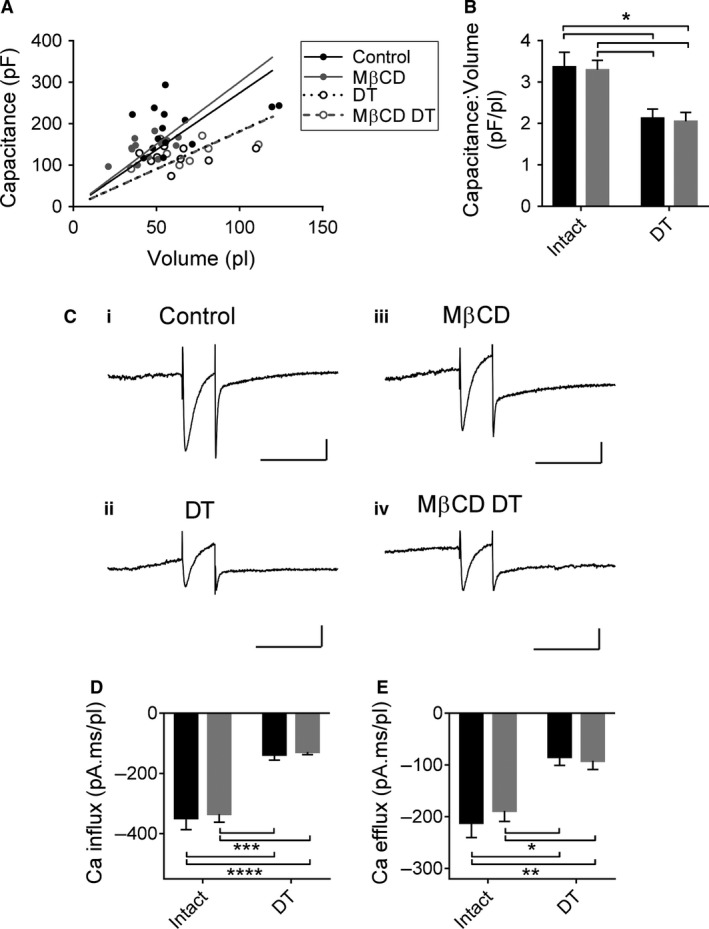
(A) Relationship between cell capacitance and volume in intact and detubulated control and M*β* CD‐treated cells. Relationships were fitted by linear regression with slopes of 2.7, 3.0, 1.8 and 1.8 for intact control and M*β* CD and detubulated control and M*β* CD, respectively. (B) Mean capacitance:volume ratio in intact and detubulated control (black bars) and M*β* CD‐treated (gray bars) cells. (C) Representative current densities in control intact (i), control detubulated (ii), intact M*β* CD‐treated (iii) and detubulated M*β* CD‐treated (iv) cells. Vertical and horizontal scale bars represent 1 pA/pF and 200 ms, respectively. (D) Mean Ca influx. (E) Mean Ca efflux. Black bars represent control and gray bars represent M*β* CD. Control intact *n* = 14/8, M*β* CD intact *n* = 13/6, control detubulated *n* = 10/7, M*β* CD detubulated *n* = 8/3, *P < 0.05, **P < 0.01, ***P < 0.001, ****P < 0.0001 with Bonferroni post hoc test.

To assess further the effect of cholesterol depletion on t‐tubules, we investigated the capacitance to volume relationship in intact and detubulated control and M*β*CD‐treated cells. Figure 2A shows the relationship between cell capacitance and cell volume in the four groups of cells. These data show that the relationship was similar in intact control and M*β*CD‐treated cells and in detubulated control and M*β*CD‐treated cells, but that cell capacitance was smaller for a given cell volume following detubulation in both control and M*β*CD‐treated cells, reflecting the loss of t‐tubular membrane capacitance. Figure 2B shows the mean capacitance:volume ratios, showing that these were similar in intact M*β*CD‐treated and untreated myocytes and in detubulated M*β*CD‐treated and untreated myocytes, but that the ratio was significantly smaller in both groups of detubulated myocytes than in the corresponding intact cells. These data suggest that cholesterol depletion has little effect on membrane capacitance and that the capacitance of the t‐tubular membrane is not different from that of the surface membrane due to cholesterol enrichment.

### Ca handling following cholesterol depletion

To investigate the role of cholesterol on Ca handling in the surface sarcolemma and t‐tubules, Ca influx (via I_Ca_) and efflux (via I_NCX_) were measured in intact and detubulated M*β*CD‐treated and untreated cells. Representative currents are shown in Figure 2C. Ca influx was not significantly affected by M*β*CD treatment and was significantly reduced by a similar amount following detubulation in both control and M*β*CD treated (*P* < 0.0001 and *P* < 0.001, respectively, with Bonferroni post hoc test, Fig. 2C,D). Similarly, Ca efflux was unaffected by M*β*CD, and detubulation caused a similar significant decrease in Ca efflux in untreated and treated myocytes (*P* < 0.01, *P* < 0.05, respectively, with Bonferroni post hoc test Fig. 2C,E). This suggests that cholesterol depletion has little acute effect on the function or distribution of either I_Ca_ or I_NCX_.

## Discussion

Previous work has suggested that cholesterol in the t‐tubule membrane could be an important determinant of t‐tubule structure and t‐tubular localization of protein function, as well as membrane capacitance, which has important implications for calculation of t‐tubule membrane fraction and current densities (see Introduction). The present work was undertaken, therefore, to test the hypothesis that the t‐tubule membrane is enriched in cholesterol compared to the surface membrane, and that this determines t‐tubule structure and function. Detubulation of ventricular myocytes was used, as described previously (Kawai et al. 1999; Brette et al. 2002), to physically and functionally detach the t‐tubules from the surface membrane, enabling us to investigate directly the effect of cholesterol depletion on t‐tubule capacitance and function, thereby obviating potential problems caused by redistribution within the membrane of the intact myocyte.

It has previously been suggested that cholesterol is important in determining t‐tubule structure (Carozzi et al. 2000; Zhu et al. 2016). The lack of effect of M*β*CD on t‐tubule structure in this study is consistent with previous work on rat myocytes (Calaghan and White 2006) but different from a recent report in mouse myocytes (Zhu et al. 2016). Although the reason for the difference is unknown, it might be due to differences in the M*β*CD treatment protocol. Zhu et al. reported disruption of t‐tubule structure following treatment with 3 mmol/L M*β*CD for 1 h, which increased with longer durations of treatment. However, concentrations of M*β*CD ≥ 3 mmol/L have been reported to cause cell damage and death in other cell types (Irie et al. 1982; Zidovetzki and Levitan 2007). The present work using filipin staining shows that 1 mmol/L M*β*CD is sufficient to deplete membrane cholesterol, without affecting t‐tubule morphology.

The lack of effect of M*β*CD on t‐tubule structure is consistent with its lack of effect on membrane capacitance in intact cells in the present study and in previous work on rat myocytes (Calaghan and White 2006). The lack of M*β*CD effect on the decrease in cell capacitance caused by detubulation suggests that t‐tubule membrane capacitance is also independent of cholesterol. These data, therefore, provide no evidence that the cholesterol content or capacitance of the t‐tubule and surface membranes are different. This is important because previous work has suggested that the t‐tubule membrane in skeletal muscle is rich in cholesterol, compared with the surface membrane (Rosemblatt et al. 1981; Hidalgo 1985), and work on artificial membranes has shown that increasing cholesterol content causes a nonlinear decrease in specific capacitance (Pásek et al. 2008); thus if t‐tubular cholesterol were high, and specific capacitance therefore low, it would result in a smaller fractional loss of capacitance than of tubular membrane following detubulation, and an underestimate of t‐tubule membrane fraction. It is not clear why cholesterol depletion does not change membrane capacitance in myocytes, since a decrease in cholesterol would be expected to decrease membrane thickness and thereby increase capacitance (which is inversely related to thickness; Levitan et al. 2000), although it is possible that cholesterol depletion has little effect on membrane thickness and/or decreases over a range at which it has relatively little effect on capacitance (Pásek et al. 2008).

Previous work has shown that I_Ca_ is located predominantly at the t‐tubules of mouse ventricular myocytes (Gadeberg et al. 2017) and has suggested that the scaffolding protein caveolin‐3 is involved in the t‐tubular localization of proteins, including Ca_v_1.2, and thus I_Ca_ (Bryant et al. 2014). Since caveolin‐3 binds to cholesterol, this implies that t‐tubular cholesterol may also be important in protein localization, and redistribution of caveolin‐3 to noncholesterol‐rich membranes in heart failure has been suggested to lead to loss of caveolin‐3 from the t‐tubules (Ratajczak et al. 2003), which could explain the redistribution of I_Ca_ away from t‐tubules observed in heart failure (Bryant et al. 2015).

The lack of effect of cholesterol depletion on I_Ca_ in intact myocytes is consistent with previous work in rat ventricular myocytes (Löhn et al. 2000; Calaghan and White 2006; MacDougall et al. 2012), and the decrease in Ca influx following detubulation in untreated cells is consistent with previous work showing that I_Ca_ is located predominantly in the t‐tubules (Gadeberg et al. 2017). Importantly, however, although some L‐type Ca channels occur within caveolae (Balijepalli et al. 2006), the observation that M*β*CD has no effect on I_Ca_ in intact cells or on the decrease in Ca influx caused by detubulation (Fig. 2D) suggests that cholesterol depletion has little acute effect on the distribution or function of either caveolar or noncaveolar I_Ca_. Similarly, the present work suggests that M*β*CD has little effect on I_NCX_ and its distribution (Fig. 2E), although efflux would be expected to reflect the amount of Ca influx assuming the cell is in steady state.

In conclusion, the data show that cholesterol depletion had no effect on t‐tubule structure, nor did it alter the capacitance:volume ratio of intact cells, or the decrease observed following detubulation, nor Ca influx or efflux, or the decreases caused by detubulation. It appears, therefore, that acute cholesterol depletion has little effect on membrane capacitance or t‐tubule structure and function and that, although the data cannot exclude it being cholesterol rich, the capacitance of the t‐tubule membrane is not different from that of the surface membrane due to high cholesterol content. Changes in membrane capacitance, if they occurred, would be expected to take place rapidly with cholesterol depletion; however, it remains possible that cholesterol depletion has long‐term effects on t‐tubule structure and function, which could contribute to the changes observed in heart failure (Bryant et al. 2015), although we are unaware of reports of loss of cellular cholesterol in failure.

## Conflict of Interest

None declared.
